# Predicted causality in decision making: the role of culture

**DOI:** 10.3389/fpsyg.2014.00479

**Published:** 2014-05-23

**Authors:** C. Dominik Güss, Bernadette Robinson

**Affiliations:** ^1^Department of Psychology, University of North Florida, Jacksonville, FLUSA; ^2^Otto-Friedrich Universität BambergBamberg, Germany

**Keywords:** dynamic decision making, problem solving, predictability, motivation, time, predicted causality, cross-cultural

## DECISIONS AND HOW THEY ARE MADE

In the wider sense, decision making is embedded in the problem-solving process and its many stages (Davidson and Sternberg, 2003; Güss et al., 2010). In the narrow sense, decision making is understood as the ability to select one of several alternatives and to act accordingly (Güss 2004). Previous research has often focused on decision making in relatively predictable environments with clear goals (e.g., expected utility theory of von Neumann and Morgenstern, 1944). In recent decades the focus has been on decision making heuristics, i.e., strategies or rules of thumb, applied in uncertain situations (e.g., Tversky and Kahneman, 1974; Simon, 1979; Gigerenzer and Gaissmaier, 2011).

Causality plays an important role in many cognitive processes – and causal cognition is itself influenced by culture (e.g., Norenzayan and Nisbett, 2000; Medin and Atran, 2004; Beller et al., 2009; Bender and Beller, 2011; for a controversial discussion of causal cognition, see Sperber et al., 1995). Causality is especially important during the decision-making process, because the decision maker has to predict what consequences specific decisions bring about before making a decision.

Causality refers here to the predicted decision options, that a specific planned action, when executed under specific circumstances, will have a specific predicted effect. This definition of causality refers to Aristotle’s causa efficiens, i.e., an action is the origin and will cause an intended effect. Our understanding of causality is a constructivist understanding, because causality refers to the causal predictions of the actor and sometimes the actor’s predicted probability of causal consequences might differ from a normative-mathematical probability of causal consequences. Predictions by actor and mathematical probability might be quite high (“As it is raining slightly, I will use the big umbrella and therefore not get wet during my walk”), but predictions by actor might be high and mathematical probability might be quite low (“when I buy a lottery ticket and use the birthdates of my family as lucky numbers, then I will win a million dollars”). Thus one could speak of predicted causality guiding the decision-making process. We are referring here to the predictions of the actor across domains.

The selection of decision alternatives is dependent on several factors such as importance, urgency, and likelihood of success (e.g., Dörner, 2008; Dörner and Güss, 2013). First, the predictions regarding decision alternatives involve the estimation of how important an alternative is. The importance is related to the human needs and the decision alternative, for example, to drink a glass of water when extremely thirsty would be more important than the decision alternative to call a friend to chat. Thus, although decision making is a cognitive process, it is related to our human needs and motivational processes.

Second, predictions regarding decision alternatives involve estimations of time and resulting urgency. If I am in my office and it is 5:30 pm, and I want to buy some groceries for the weekend and I know the store closes at 6:00 pm, and I know it takes me 15 min to get to the store, then the decision alternative “check and respond to emails” is perceived as less urgent (if the time estimation to check and respond to emails is longer than a few minutes which is usually the case).

Third, predictions regarding decision alternatives involve estimations of how likely it is that the predicted consequences actually happen. I know 15 min is the time I need to go to the store and I know I need an hour to check my emails and to respond to them. This predicted likelihood of success is dependent on one’s competence: first the epistemic competence, i.e., the fact knowledge and experiential knowledge of the past; and second, the general competence, i.e., an estimation of one’s ability to act successfully in the given situation (Dörner 2008). High general competence is reflected in high predicted likelihood of success for decision alternatives (“I can do this”). In other words, one believes in oneself and that translates into one’s ability to deal with situations successfully.

Judging importance, urgency, and likelihood of success for decision alternatives can occur either automatically or deliberately, i.e., unconsciously or consciously. Automatically means that based on previous experiences in similar situations, the predictions and their results are known and attributed to the current situation. Often certain cues in the current situation trigger the memory of similar situations and connected with those the successful actions in those situations which can then be applied in the current situation (e.g., recognition-primed decision making according to Klein, 2008).

If the current situation is a novel situation, then deliberations about possible consequences of decision alternatives are more likely to take place. The novel situation requires deliberate thinking and predicting possible causal developments of decision options.

## CULTURAL INFLUENCES ON THE PREDICTED CAUSALITY DURING DECISION-MAKING

The brief discussion of decision making and the variables influencing the selection of a decision alternative suggest ways in which culture influences the decision-making process and in which cultures could differ. Culture can be understood as implicit and explicit knowledge – including knowledge about how to make decisions and what decisions to make under what circumstances – shared by a specific group of people and transmitted from generation to generation (e.g., Smith et al., 2006). According to action theory and sociocultural theories, this knowledge is acquired during social interactions (Vygotsky, 1978) in a specific cultural, social, and historical context (Cole, 1996) which offers similar opportunities for learning (e.g., Lave, 1991).

## IMPORTANCE – MOTIVATIONS

Previously we have stated that the estimation of decision alternatives’ importance is related to needs. Although one can assume the universality of some human needs (Maslow, 1954), for example the existential needs and the needs for sexuality, affiliation, certainty, and competence (Dörner, 2008) or the needs for autonomy, competence, and relatedness as outlined in self-determination theory (Ryan and Deci, 2000), it is very likely that the importance of these needs varies across cultures. Church et al. (2013), for example, tested self-determination theory in eight cultures and found that Asian participants (Japan, China, Malaysia, and the Philippines) showed lower need satisfaction of competence and autonomy compared to American participants (United States, Mexico, and Venezuela). Additionally, research on individualism and collectivism has shown that for members of collectivist cultures, social and relational aspects of decisions might be more important compared to members from individualistic cultures (e.g., Güss, 2004).

Thus, the cultural importance of certain needs triggers different importance ratings for decision alternatives related to these needs.

## URGENCY – TIME

Cultures encourage their members to develop different expectations regarding time and the future; not only the content of future developments, but also their structure (Güss, 2013). Structural differences can refer to the breadth and width of future expectations. Does the development of decision alternatives and their related causal predictions concern the near future or the far future? Do decision makers develop one decision alternative or several?

Stable as opposed to unpredictable cultural environments, are those cultural environments in which social, political, economic, and/or climate-geographic changes are minimal and therefore allow their citizens long-term planning and decision making (Strohschneider and Güss, 1999). In relatively unpredictable cultural environments, it is not adaptive to develop predictions that reach far into the future. The predictions about possible likelihoods of events would be too difficult to make, for example during times of inflation. Yet, it is adaptive to develop several short-term plans. In relatively stable cultures, it is more adaptive to develop predictions and to make decisions that reach far into the future. Evidence for this argument can be found in the following cross-cultural studies on dynamic decision making and planning.

German, U.S., Indian, Filipino, and Brazilian participants were presented with the dynamic, non-transparent task Coldstore (Güss and Dörner, 2011). Participants attempted to regulate a broken thermostat which was simulated on the computer and to maintain an ideal temperature during this task. The thermostat does not react right away, but is time-delayed. When it is turned up, it takes a little while for the temperature to heat up; and when it is turned down, it takes a while for the temperature to cool down.

German and U.S. participants showed adaptor-type decision making more often than Indian, Filipino, and Brazilian participants who showed more oscillator-type decision making. Adaptor-type decision making means observing long-time intervals of changes in the system and adjusting slowly. Oscillator-type decision making means reacting to the momentary situation and regulating the temperature from one extreme to the other extreme without considering adequately what happened before and without taking possible predicted developments into consideration.

Regarding planning, researchers investigated these differences using daily life scenarios in Brazil during a time of extremely high inflation and in Germany during relatively stable economic conditions. German plans were longer and had more decision alternatives compared to Brazilian plans. Interestingly, Brazilian compared to German participants were more optimistic about the potential results of their decisions (e.g., Güss, 2000). Thus decision making was adapted to the conditions of the cultural context.

## LIKELIHOOD OF SUCCESS – EPISTEMIC AND GENERAL COMPETENCE

Prediction of consequences and their likelihood of success are partly based on the epistemic competence, i.e., world knowledge in general and on specific domain knowledge in particular which the decision maker has accumulated over time. World knowledge is highly dependent on culture such as what we learn when we grow up.

Heuristics are a part of this epistemic knowledge which is acquired during socialization. Such heuristics can differ between cultures. Several experiments have shown, for example, that Chinese participants when confronted with uncertain and contradicting materials preferred a compromise. European Americans, however, tried to choose one correct position (Peng and Nisbett, 1999). Thus the Chinese learned and applied a “find a middle way” heuristic, whereas the European Americans learned and applied a “find the right way” heuristic.

Estimating the likelihood of success is also based on general competence, i.e., the estimation of one’s abilities to deal successfully with the current situation. This general competence also varies across cultures (see also cross-cultural differences in self-efficacy, e.g., Scholz et al., 2002). Many studies have shown, for example, that Chinese students outperform U.S. students on international math tests (e.g., Beaton et al., 1996). One explanation for this finding is that Chinese “students perceived controllable causes, particularly effort, to play a greater role in performance outcomes than did their American peers” (Tuss et al., 1995, p. 408). Also the Chinese mothers viewed effort as the main cause for low math performance, whereas American mothers attributed low performance to other causes as well (Hess et al., 1987). Thus, the actual math performance might be related to a higher feeling of general competence (because effort is controllable) in the Chinese compared to the American sample.

## CONCLUSION

**FIGURE 1:**
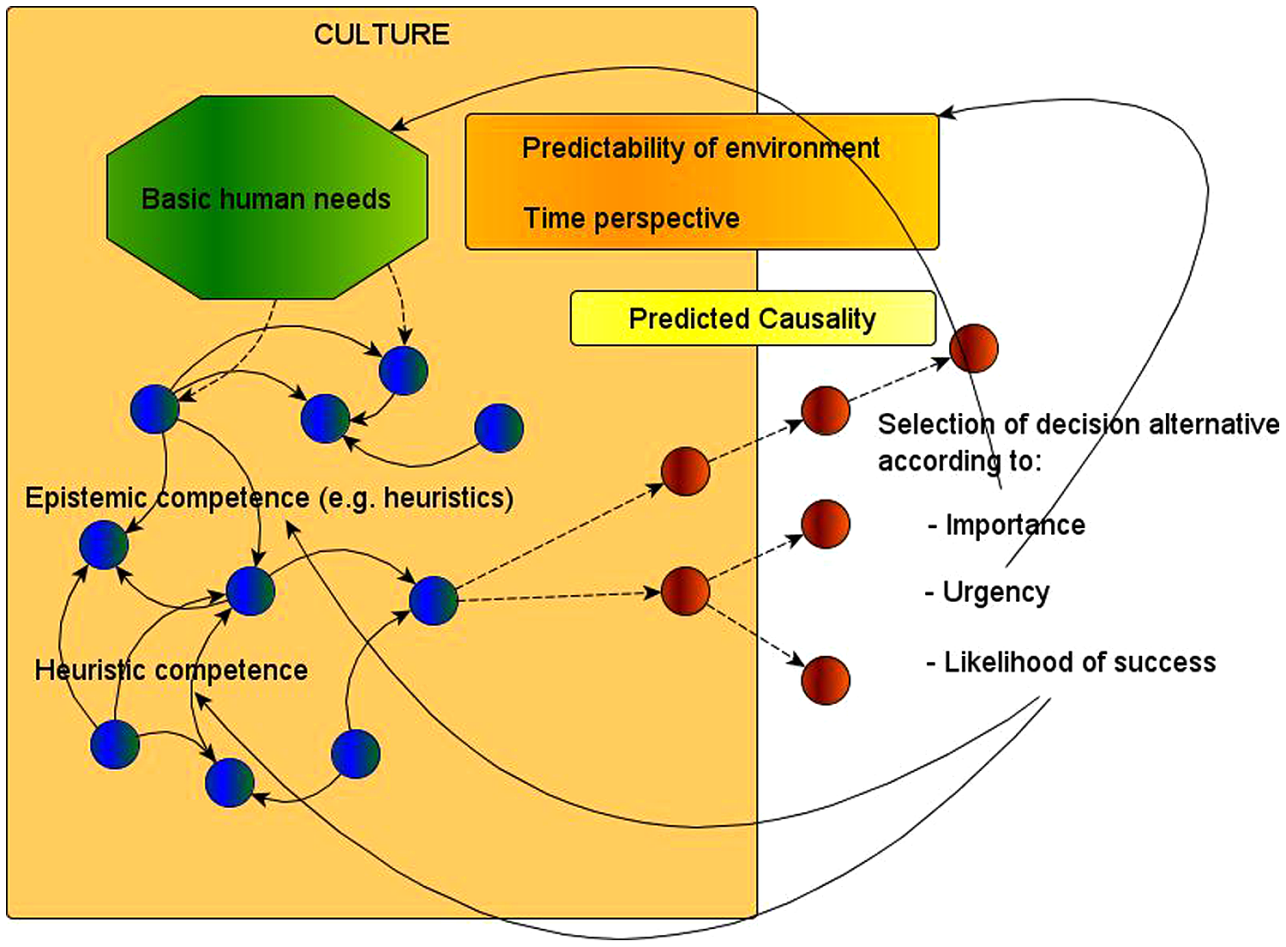
**FIGURE 1. Factors influencing predicted causality, decision making, and the role of culture.** The knowledge structure is shown as a simplified neural network with interconnected neurons in blue color. From the node representing the current situation two dashed arrows go to two predicted situations (represented in brown dots). The first one predicts two further situations as probable linear consequences when certain actions take place (represented by dotted arrows). The second one predicts two different further developments when specific actions are taken. The selection of one decision alternative then depends on importance, urgency, and likelihood of success. Importance refers to the strength of a specific need. Urgency refers to predicted time needed to execute a decision. Likelihood of success refers to existing knowledge, i.e., epistemic competence, and heuristic competence, i.e., the estimation of one’s abilities to deal successfully with the current situation. Cultures differ regarding importance, urgency, likelihood of success, and predicted causal developments.

The main argument of this paper is that decision making involves causal predictions about possible future developments and that decision making involves estimation of importance, urgency, and decision alternatives’ likelihood of success (see **Figure 1**). We then presented results from cross-cultural research showing that these processes differ among cultures and that culture highly influences decision making. These cross-cultural differences in decision making highlight the embeddedness of decision making within a specific eco-cultural historical context. To put it in extreme but very realistic terms, every decision is a cultural decision.

## Conflict of Interest Statement

The authors declare that the research was conducted in the absence of any commercial or financial relationships that could be construed as a potential conflict of interest.
